# Impact of self-management interventions on stable angina symptoms and health-related quality of life: a meta-analysis

**DOI:** 10.1186/1471-2261-14-14

**Published:** 2014-02-01

**Authors:** Michael McGillion, Sheila O’Keefe-McCarthy, Sandra L Carroll, J Charles Victor, Tammy Cosman, Allison Cook, John G Hanlon, E Marc Jolicoeur, Noorin Jamal, Robert McKelvie, Heather M Arthur

**Affiliations:** 1 Faculty of Health Sciences, McMaster University, 1280 Main Street West, Room HSC 2J20A, Hamilton, ON L8S 4K1, Canada; 2 University of Toronto, 155 College Street, Toronto, ON M5T 1P8, Canada; 3 Hamilton Health Sciences, 711 Concession Street, Hamilton, ON, N3L 2Y6, Canada; 4 Montreal Heart Institute, 5000, Bélanger Street, Montréal, QC H1T 1C8, Canada

**Keywords:** Stable angina, Self-management, Health-related quality of life

## Abstract

**Background:**

Chronic stable angina (CSA) has a major negative impact on health-related quality of life (HRQL) including poor general health status, psychological distress, and inability to self-manage.

**Methods:**

We used meta-analysis to assess the effectiveness of self-management interventions for improving stable angina symptoms, HRQL and psychological well-being. Nine trials, involving 1,282 participants in total, were included. We used standard inverse-variance random-effects meta-analysis to combine the trials. Heterogeneity between trials was evaluated using chi-square tests for the tau-squared statistic and quantified using the I^2^ statistic.

**Results:**

There was significant improvement in the frequency of angina symptoms (Seattle Angina Questionnaire [SAQ], symptom diary) across trials, standardized mean difference (SMD): 0.30 (95% Confidence interval [CI] 0.14, 0.47), as well as reduction in the use of sublingual (SL) nitrates, SMD: -0.49 (95% CI -0.77, -0.20). Significant improvements for physical limitation (SAQ), SMD: 0.38 (95% CI 0.20, 0.55) and depression scores (Hospital Anxiety and Depression Scale), SMD: -1.38 (95% CI -2.46, -0.30) were also found. The impact of SM on anxiety was uncertain due to statistical heterogeneity across trials for this outcome, I^2^ = 98%. SM did not improve other HRQL dimensions including angina stability, disease perception, and treatment satisfaction.

**Conclusions:**

SM interventions significantly improve angina frequency and physical limitation; they also decrease the use of SL nitrates and improve depression in some cases. Further work is needed to make definitive conclusions about the impact of SM on cardiac-specific anxiety.

## Background

Chronic stable angina (CSA) is a primary symptom of coronary artery disease (CAD) characterized by the predictable occurrence of pain or discomfort in the substernal and adjacent areas of the chest [1]. Symptomatology can vary in terms of severity (Canadian Cardiovascular Society [CCS] class) and may include anginal equivalents such as nausea, breathlessness upon exertion, and/or fatigue [1]. Classic presentation of CSA is featured by reversibility of symptoms and repetitiveness of angina episodes over time, typically months or years [2]. As CAD survival rates increase, the global prevalence of CSA is also rising. Prevalence estimates suggest that CSA affects 3.8% of Americans aged 20 and over (6 million) and 1.9% of Canadians over the age of 12 (n = 483,000) [3]. In Scotland, CSA is prevalent in 2.6% of the general population, with 28 per 1000 men and 25 per 1000 women being affected, respectively [4].

The public health burden of CSA is considerable. Cumulative data indicate that those with CSA are among the most debilitated across several chronic illness populations including sciatica, arthritis, low back pain, and stroke [5]. With respect to CAD in particular, a recent retrospective cohort study (n = 1,609) found that those with CSA alone were more physically incapacitated than those with a history of previous myocardial (MI) infarction and/or revascularization procedures [6]. Aside from physical limitation, CSA also imposes significant psychological impact on health-related quality of life (HRQL). Many patients report heart-focused attention and anxiety, fear of death, depression, impaired role functioning, and poor sense of general health and well-being [7-9]. This high level of symptom burden has major financial consequences both at patient and health care system levels. At the patient level, McGillion et al. estimated the cost of CSA-related disability— expressed as direct out of pocket, indirect and system costs— at $19,209 per annum; this estimate was likely to be conservative due to reliance on self-report measures [10]. In the UK, the direct system costs of angina symptom management including prescriptions, hospital admissions, outpatient referrals, and investigational procedures, were estimated in 2000 at ₤669, 000, 000, accounting for 1.3% of total National Health Service expenditure [11].

Increasing attention has being given to angina self-management [SM] interventions as an adjunctive means to offset the symptom-related burden of CSA. The goal of SM is to prevent or slow chronic illness symptom-related disability and restore functioning and life roles to optimal levels. Typical SM programs provide educational materials and coaching methods to achieve positive changes in knowledge and behaviour for effective disease self-management. Program models vary but are commonly grounded in social cognitive and learning theories, such as Bandura’s Self-Efficacy Theory [12], which targets the following mediators of individual performance: a) perceived self-efficacy or confidence, and b) perceived effectiveness of learned behaviours to achieve desired health outcomes.

Published randomized clinical trials (RCTs) of angina SM programs to date have targeted improvements in angina symptom profile and related SL nitrate use, functional aspects of HRQL, and psychological well-being [13-21]. Our aim was to examine comprehensively the overall effectiveness of SM for improving these outcomes.

## Criteria for selection of studies included in this review

### Types of studies

We included all published and unpublished RCTs of SM interventions delivered by a trained professional or lay-trained facilitator in individual or group formats, with parallel designs; length of follow up period varied. Non-randomized studies and single-group design studies were excluded.

### Types of participants

Adult outpatients of all ages with ischemic heart disease (IHD) and Canadian Cardiovascular Society (CCS) Class I – IV angina, reporting stable symptoms for at least 3 months, were included.

### Types of interventions and controls

Interventions employing a combination of cognitive and behavioural angina self-management techniques were included such as: supportive coaching, anxiety and stress management or counselling, incremental exercise program, nutrition planning, medication review, relaxation training, and energy conservation. Controls received routine or usual care and were not exposed to the intervention during the study period.

### Types of outcome measures

1. Angina symptom profile including angina frequency and stability and related SL nitrate use

2. HRQL dimensions including physical limitation, disease perception, and treatment satisfaction

3. Psychological well-being, reflected by anxiety and depression

## Methods

### Search for identification of studies

We searched the Cochrane Central Register of Controlled Trials, MEDLINE, PubMed, CINHAL, EMBASE, Proquest Dissertation Abstracts, Psychinfo and HealthStar, Jan. 1990 – Aug. 2013, using combinations of key medical subject heading (MeSH) terms including chronic stable angina, stable angina, self-management, self-care, patient education randomized controlled trials, and clinical trials. In addition, trial registers including the World Health Organization (WHO) International Clinical Trial Registry Platform, clinicaltrial.gov, the ISRCTN register, and *Meta*Register were searched for relevant ongoing or completed studies with potential publication. We also conducted hand searches of relevant journals and secondary references, as well as proceedings of international conferences; experts were also consulted for additional sources. No restrictions were applied with regards to language, sample size or length of follow-up. Our search strategy was critiqued and replicated by an information specialist to ensure comprehensiveness.

### Final selection of trials

Five reviewers (MM, JCV, SC, AC, HA) reached consensus on trials to be included in this analysis by reviewing the titles, abstracts and reports of trials according to the inclusion criteria specified *a priori*; individual trial results were not considered during this process.

### Data extraction and appraisal and reporting of methodological quality

Four reviewers (MM, SOM, SC, NJ) participated in independent quality assessment and extraction of process and outcome data from each new trial according to a standardized extraction format we have used in other reviews [22-24]. Methodological quality of included trials was appraised via standard Cochrane criteria for risk of bias assessment [25] including generation of randomization sequence; allocation concealment; blinding of participants, personnel and outcome assessors (detection bias); standardized intervention delivery and presence of co-intervention (performance bias); reliability and validity of measurement instruments (insensitive measurement bias); response rate (RR) and attrition (attrition bias); and selective reporting (reporting bias). Propensity for selection bias was also assessed. Reported outcome data were taken directly from included published trial reports. The report on the quality of methods presented in this paper is compliant with standards set forth in the Preferred Reporting Items for Systematic Reviews and Meta-Analyses (PRISMA) statement [26].

### Data synthesis and analysis

All outcomes examined were continuous in nature. For all relevant outcome data, standardized mean differences (SMD) and 95% confidence intervals were calculated using RevMan 5.1.7® software [27]. SMDs were determined using differences in change over baseline post- intervention across treatment groups, divided by the pooled standard deviation. If change over baseline was unavailable, differences in mean values at the end of the treatment period were used. If some of the required data were unavailable we used approximations based on graphic output. For studies reporting only means and interquartile ranges, means and standard deviations were estimated [28,29]. A SMD of 0.20 standard deviation units was considered a small difference between treatment and control groups, a SMD of 0.50 a moderate difference, and 0.80 a large difference [30]. We used standard inverse-variance, random-effects meta-analysis to combine the trials.^31^ Heterogeneity between trials was evaluated using chi-square tests for the tau-squared statistic, quantified using the I^2^ statistic [31], which describes the percentage of variation across trials attributable to heterogeneity rather than chance. I^2^ values of 25%, 50%, and 75% may be considered as indicators of low, moderate, and high heterogeneity [32], although this has been shown to depend on the size and number of trials included [33]. Where significant heterogeneity was found, we conducted sensitivity analyses removing studies, such as those with estimated mean values or those of lower methodological quality, to determine factors related to the heterogeneity and the effect on the pooled outcome.

### Description of studies

Nine trials [13-21], conducted in 7 countries and published between 1994 and 2012 met the criteria for inclusion in this review. Sample sizes ranged from 29 [13] to 452 [17]. Eight of the included trials reported use of an isolated SM intervention with components designed to enhance patients’ perceived confidence and skills to manage symptoms [13-17,19-21]. Control groups received usual medical and/or nursing care as described; no controls were exposed to the intervention during the study period.

Five trials [13,14,16,17,19] tested small-group interventions (6–15 patients) employing varying combinations of educational materials, planned exercise, and cognitive-behavioural techniques targeted at lifestyle and symptom self-management, relaxation training or stress reduction, or enhancement of physical activity. Intervention duration, format, and process varied. Four trials tested interventions with content similar to the group-based interventions but on an individual basis [15,18,20,21]. All trials included baseline assessment of participant characteristics and outcomes prior to randomization. Most trials used symptom diaries to measure angina symptom profile and related SL nitrate use; objective measures of ischemia were less often used. Subjective measures were also most often used to examine HRQL outcomes and aspects of psychological well-being. The maximum length of follow up for data pertinent to this review (i.e., pertinent to the outcomes we examined per se) was 24 weeks following baseline [15,20,21].

### Risk of bias in included studies

**Figure 1:**
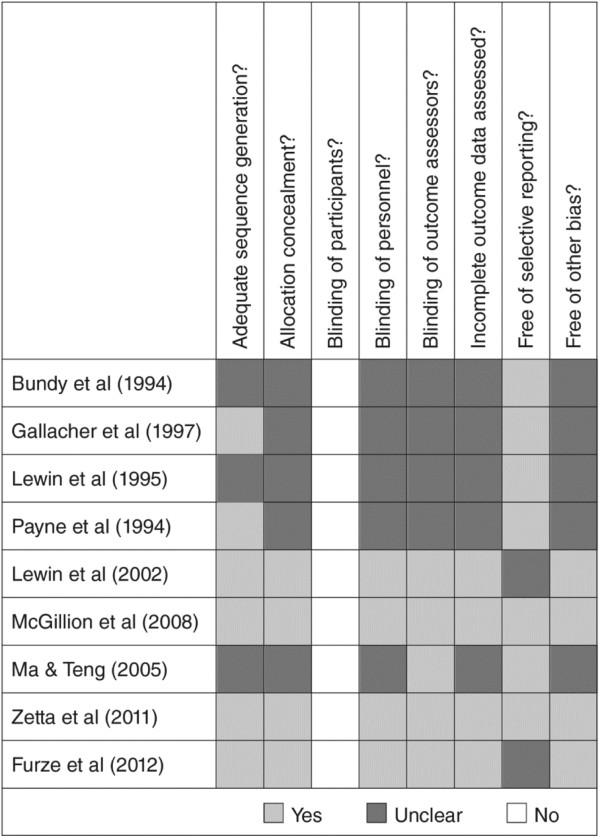
Risk of bias assessment of included trials expressed as yes/no/unclear.

**Figure 2:**
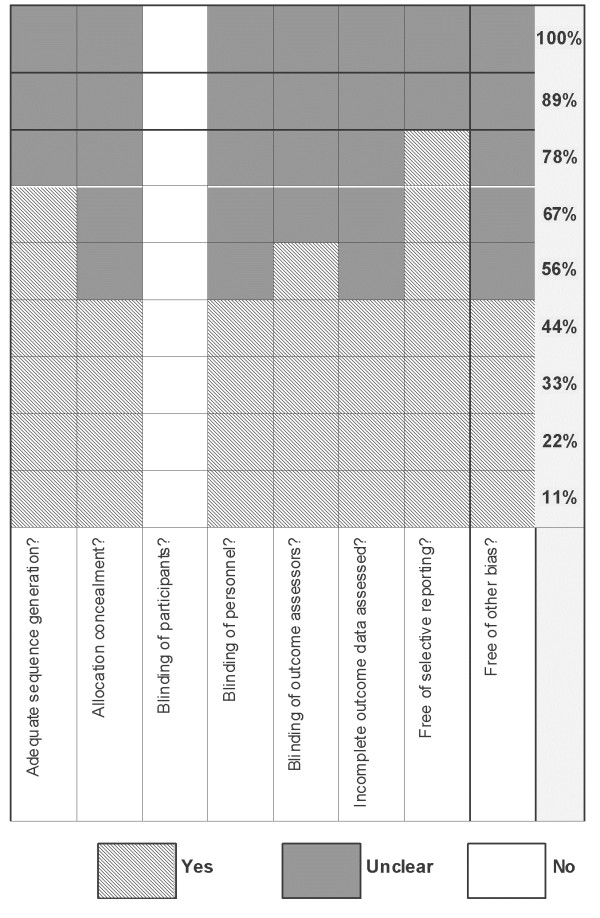
Risk of bias assessment of included trials expressed as percentage.

Details of our risk of bias assessment are provided in Figures 1 and 2. The methodological quality of the nine trials ranged from low [13,14,16-18] to high [15,19-21]. Six of the 9 trials (67%), adequately described the randomization process with respect to sequence generation and 4 trials clearly reported allocation concealment procedures (44%). Blinding of healthcare personnel caring for participants (not directly involved in the trials) occurred in 4 (44%) of the 9 included trials; no trial blinded participants given that SM interventions are socially-based. Blinding of outcome assessors was clearly outlined in 5 trials (56%) and incomplete data were addressed in 4 trials (44%). Seven trials (78%) reported on all pre-specified outcomes at all follow up time points and were free of selective reporting bias. Loss to follow up rates ranged from 0% - 19%. The results of two trials were applicable to men only [16,17], thereby limiting generalizability.

## Results

Nine trials, involving 1282 CSA patients in total, were included. It was not possible to include results from two trials [17,18] in any pooled estimates of effect due to the heterogeneity of their respective measures and analyses. All results pertain to pooled short-term effects, given the maximum length of follow-up of 24 weeks.

### Angina symptom profile

Angina symptom profile was examined with respect to angina frequency and related SL nitrate use, and angina stability. Three trials [13,15,16] reported on angina frequency expressed solely as counts of angina attacks in the previous week while 4 trials [15,19-21] reported changes in angina frequency via the Seattle Angina Questionnaire (SAQ) angina frequency subscale (with our without the additional use of symptom diaries). Therefore, we performed subgroup meta-analyses, separating the studies according to form of outcome assessment. In studies where both data collection methods were used, preference was given to data gleaned by the SAQ due its well-established psychometric properties [34]. We then pooled for overall effect.

**Figure 3:**
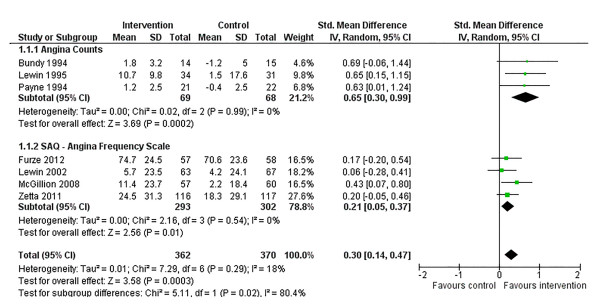
Comparison of SM versus usual care, outcome angina frequency.

Following SM training, there was a significant improvement in angina frequency across studies, SMD: 0.30 (95% confidence interval [CI] 0.14, 0.47. p = 0.0003) (Figure 3). This significant finding was also seen when trials were grouped by form of outcome assessment, although the effect size was significantly larger across trials measuring counts of angina episodes per week, chi-square (*χ*^2^): 5.11 (p = 0.02) (Figure 3). There was no significant heterogeneity of variances in the overall pooled analysis or in either subgroup.

**Figure 4:**
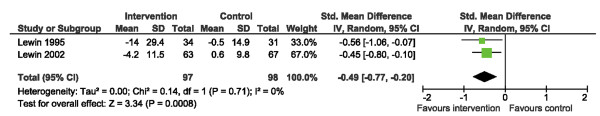
Comparison of SM versus usual care, outcome SL nitrate use.

SL nitrate use was reported in two trials [14,15]. SM interventions resulted in a significant reduction of SL nitrate usage, SMD: -0.45 (95% CI -0.77, -0.20. p < 0.001) (Figure 4). No significant heterogeneity was found.

**Figure 5:**
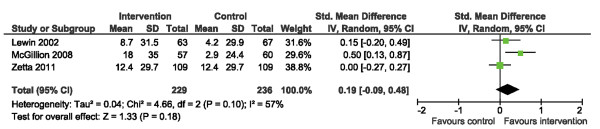
Comparison of SM versus usual care, outcome angina stability (AS).

Three trials [15,19,21] reported on angina stability using the stability subscale of the SAQ. No significant differences in angina stability scores were found (Figure 5). There was mild heterogeneity across studies, I^2^ = 57%, but this was not statistically significant.

### Health-related quality of life

**Figure 6:**
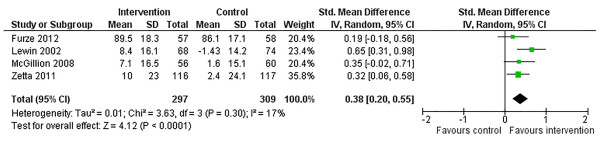
Comparison of SM versus usual care, outcome physical limitation (PL).

**Figure 7:**
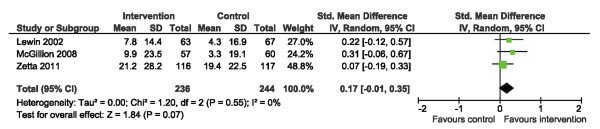
Comparison of SM versus usual care, outcome disease perception (DP).

**Figure 8:**
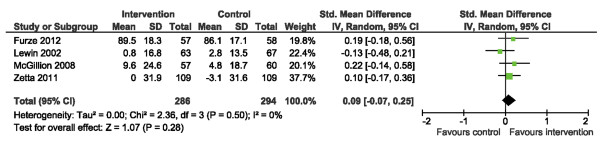
Comparison of SM versus usual care, outcome treatment satisfaction (TS).

We examined HRQL outcomes according to the SAQ physical limitation, disease perception, and treatment satisfaction subscales [34]. Of the 4 trials reporting SAQ physical limitation scores [15,19-21], one reported post-intervention scores only while the other 3 reported change from baseline. Use of SMDs allowed the 4 studies to be pooled (Figure 6). Across trials, a significant improvement in physical limitation scores was observed post-intervention, SMD: 0.38 (95% CI 0.20, 0.55. p < 0.0001); statistical heterogeneity was negligible, I^2^ = 17%. Three [15,19,21] and 4 [15,19-21] trials reported disease perception and treatment satisfaction scores, respectively (Figures 7 and 8). No significant improvements in disease perception or treatment satisfaction were found and there was no significant heterogeneity of variances across studies for these outcomes.

### Psychological well-being

**Figure 9:**
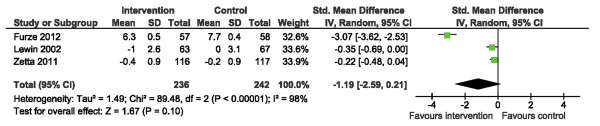
Comparison of SM versus usual care, outcome anxiety (HADS-A).

**Figure 10:**
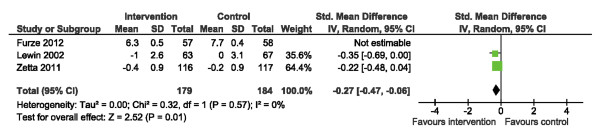
**Sensitivity analysis: comparison of SM versus usual care removing the influence of data from Furze et al.**[20]**, outcome anxiety (HADS-A).**

Data pertaining to psychological well-being [15,20,21] were amenable to pooling using the Hospital Anxiety and Depression Scale (HADS) [35]. We initially found no significant difference in anxiety scores (HADS-A), yet there was considerable heterogeneity across studies, I^2^ = 98% (Figure 9). Sensitivity analysis, via removal of the trial by Furze et al. [20] with the widest CI, improved heterogeneity (I^2^ = 0%) and suggested an overall significant reduction in HADS-A scores following SM training (Figure 10), SMD: -0.27 (95% CI -0.47, -0.06. p = 0.01).

**Figure 11:**
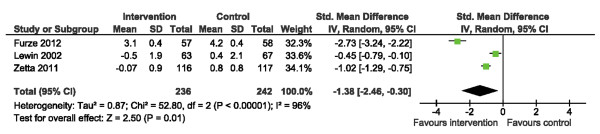
Comparison of SM versus usual care, outcome depression (HADS-D).

With respect to depression (HADS-D), there was a significant difference in HADS-D scores following SM training SMD: -1.38 (95% CI -2.46, -0.30. p = 0.01) (Figure 11). However, there was considerable heterogeneity across studies (I^2^ = 96%) which was statistically significant. Following sensitivity analyses, no removal of a single study improved heterogeneity such that I^2^ was <80%. Regardless, we observed significant reduction in HADS-D under all scenarios, suggesting that the positive impact of SM training on depression is stable.

## Discussion

In this review we have appraised and summarized the results of 9 trials of interventions for angina self-management, conducted across 7 countries, in a variety of outpatient clinic settings. All trials reviewed included patients with stable angina, characterized by CCS class I – IV symptoms. The methodological quality of the trials ranged from low to high, with 5 trials blinding outcome assessors and 4 trials blinding health care personnel directly caring for participants (outside of direct trial-related activities).

The first outcome examined was angina symptom profile including angina frequency and related SL nitrate use, as well as stability of symptoms. Symptom frequency was expressed as either weekly count of angina episodes or in the form of standardized scores via the angina frequency subscale of the SAQ. Consistent with a prior, smaller scale review [23], we found that SM interventions significantly improve the frequency of symptoms in the short term and reduce usage of SL nitrates. We also observed a SMD in overall angina frequency of .31 (95% CI 0.51, 0.47), suggesting a lesser magnitude of effect on this outcome than what has been found previously [23]. This difference is likely due to the fact that our overall pooled estimate included trials which employed the SAQ to measure angina frequency, as opposed to others which employed diaries to capture counts of weekly angina episodes. In our subgroup analysis, the effect size for angina frequency was significantly larger across trials which reported angina counts only. Some dilution of the overall pooled effect was to be expected given that the SAQ angina frequency subscale [34] captures how many times, on average, one has had angina over a recall period of 4 weeks, expressed in terms of ranges (e.g. 1–2 times per week). Therefore, trials employing this measure are not likely to capture true angina frequencies, but rather, important changes in symptom trends [34,36].

More recent trials [19-21] in this review did not evaluate reductions in SL nitrate usage. This may reflect current understanding that such reductions are not always clinically desirable [37,38]. A noted gap in SL nitrate-related education across a number of SM trials we reviewed was the omission of education about prophylactic nitrate use, which can significantly reduce exertional angina [37,38]. This gap may be reflective of widespread usage of long-acting nitrate formulations across clinical settings [37].

The final component in the angina symptom profile was angina stability. No significant differences in SAQ angina stability scores were found across the three trials reporting on this outcome and the observed statistical heterogeneity across studies was not significant. This finding is likely explained by variable levels of effectiveness across interventions to optimize participants’ levels of activity. The angina stability subscale measures changes in the frequency of angina at patients’ most strenuous levels of activity [34]. Achievable levels of physical exertion would have varied widely given the divergence in scope and format of SM interventions across trials reviewed.

Health-related quality of life outcomes we examined were according to the SAQ physical limitation, disease perception, and treatment satisfaction subscales. SM training yielded significant improvement in angina-induced physical limitations, but did not improve treatment satisfaction. The SM interventions across the 4 trials contributing to the pooled effect on physical limitation [15,19-21] included the Chronic Angina Self-Management Program (CASMP) [19] and the Angina Plan [15,20,21]. The effectiveness of these interventions to improve physical limitation scores is likely explained by their strong grounding in self-efficacy theory [12] and supportive approaches to goal setting which target realistic, incremental improvements in functional status.

There was no heterogeneity of variances across trials in treatment satisfaction scores and lack of improvement in this outcome, across trials as well as our pooled analysis, is likely driven by the psychometric properties of the SAQ treatment satisfaction scale [34]. This scale is comprised of 3 items oriented toward patient satisfaction with physician care [34]. Interventions of included trials were delivered by either nurses [15,19,21] or lay facilitators [20], not physicians. Moreover, relatively short-term duration follow up data collection (maximum: 24 weeks) across trials would not have allowed sufficient time for improved satisfaction with physician-related care in the clinical setting. At the individual level, care delivery by more than one physician could also have introduced loss of precision in the measurement of treatment satisfaction.

With respect to disease perception, SM interventions appeared on the whole, less effective for improving this outcome. Previously, we did find a positive pooled effect for disease perception, but CIs were wide signaling caution in our interpretation of the result. The trial by Zetta et al. (n = 233) [21] carried 48.8% of the weight within this current analysis and their SM intervention had no significant impact on disease perception scores. The limited ability of SM training to improve these scores to date may be a function of heavy disease-related burden among angina sufferers; it may also be an artifact of short-term follow up across studies, given that meaningful changes in perceived disease status can take time. In this case, measurement is not likely the issue. The SAQ disease perception subscale is well-established as sensitive to change and is also highly correlated with generic self-report measures of general health and vitality [36].

The final outcome of this review was psychological well-being. The 3 trials included in our analysis [15,20,21] reported on anxiety and depression using the HADS [35]. We found the positive, significant impact of SM training on depression (HADS-D) to be stable, despite our inability to reduce statistical heterogeneity below I^2^ = 80% via sensitivity analyses. This finding is encouraging given that depression is predictive of a number of poor cardiac-related outcomes [39]. Each of the trials contributing to our pooled HADS estimates employed the Angina Plan [15,20,21]. The positive result speaks to the importance of the design of this intervention with respect to the inclusion of individualized attention and supportive counseling. The impact of group-based SM [19] on HADS-D has not been examined in primary trials.

Unlike depression, the impact of SM training on anxiety is less certain. Initially, we found no significant results. Once we removed the trial with the widest CI [20] our sensitivity analysis demonstrated a significant yet small overall reduction in HADS-A scores, SMD -0.27. Intervention structure and process were homogenous among included trials and therefore cannot explain our result. Given the heterogeneity observed in HADS scores, a single trial, regardless of size, is unlikely to confirm a significant pooled effect of SM training on anxiety; we estimate that approximately 6 or more trials, with a total sample size of 100 or more, would be required to establish a significant intervention effect with 80% conditional power (assuming a small effect size as observed in this sensitivity analysis). It is plausible that non-cardiac related anxiety contributed to an overall lack of measurement precision. Future trials should therefore employ a cardiac-specific measure of anxiety to make more definitive conclusions about the ability of SM to impact anxiety positively.

### Study limitations, summary and implications

As Le Lorier et al. [40] have argued about sources of bias in meta-analyses, an important limitation of this review is that our conclusions may be subject to the same potential for bias as the smallest trials included [13,16]. Yet, threats to validity are likely offset by our rigorous approach to risk of bias assessment as well as formal evaluation of the impact of statistical heterogeneity on outcomes. Our results for psychological outcomes must be interpreted with caution due to heterogeneity. The methodological quality of the included trials also ranged from low to high. While it is not possible to blind participants in socially-based interventions, blinding of outcome assessors was addressed in just 5 of the 9 trials examined.

## Conclusions

In summary, SM interventions appear to be effective for improving the frequency of angina symptoms and related physical limitation. SM training also appears to have an overall positive impact on depression scores, which has not been found previously [22,23]. Interventions in this review were delivered in either group-based or individual-based formats; intervention duration and intervener credentials also varied. The ideal intervention design to yield maximal and replicable long-term benefit for patients remains unknown. Future work is needed to examine the relative effectiveness of successful intervention designs in the context of robust, multi-site trials with long-term follow up. Clarity is also needed regarding the ability of SM interventions to reduce cardiac-specific anxiety for people living with CSA.

## Competing interests

The authors declare that they have no competing interests.

## Authors’ contributions

MM, SC, JCV, SOM, and HMA designed this systematic review and oversaw all aspects of the methodology. MM, SOM, SC and HMA drafted first version of the manuscript. MM, JVC, SC, AC, TC, and HMA reviewed all abstracts and came to consensus on included studies. MM, SOM, SC, and NJ participated in data extraction and conducted the risk of bias assessment of included trials. JCV, JH, and EMJ piloted tested and revised the meta-analytic strategy. JCV conducted the final data analyses. RM, HMA, EMJ, and SC revised the discussion section substantively. All authors reviewed the manuscript and contributed to revising and approving the content of the final version for submission for publication.

## Pre-publication history

The pre-publication history for this paper can be accessed here:

http://www.biomedcentral.com/1471-2261/14/14/prepub

## References

[B1] Abrams J Thadani U Therapy of stable angina pectoris: the uncomplicated patient Circulation 2005 112 e255 e259 10.1161/CIRCULATIONAHA.104.526699 16216965

[B2] Abrams JA Chronic stable angina N Engl J Med 2005 352 2524 2533 10.1056/NEJMcp042317 15958808

[B3] Chow CM Donovan L Manuel D Johassen H Tu JV Tu CJ, Ghali W, Brien S Regional variation in self reported heart disease prevalence in Canada CCORT Canadian Cardiovascular Atlas: A Collection of Original Research Papers Published in the Can J Cardiol 2006 2 Toronto: Pulses Groups Inc. and the Institute for Clinical Evaluative Sciences 23 29

[B4] Murphy NF Simpson CR MacIntyre K McAlister FA Chalmers J McMurray JJV Prevalence, incidence, primary care burden, and medical treatment of angina in Scotland: age, sex and socioeconomic disparities: a population-based study Heart 2006 92 1047 1054 10.1136/hrt.2005.069419 16399851 PMC1861126

[B5] Lyons RA Lo SV Littlepage BNC Comparative health status of patients with 11 common illnesses in Wales J Epidemiol Community Health 1994 48 388 390 10.1136/jech.48.4.388 7964339 PMC1059989

[B6] Buckley B Murphy AW Do patients with angina alone have a more benign prognosis than patients with a history of acute myocardial infarction, revascularization or both? Findings from a community cohort study Heart 2009 95 461 467 10.1136/hrt.2008.146944 18669551

[B7] McGillion M Watt-Watson J LeFort S Stevens B Positive shifts in the perceived meaning of cardiac pain following a psychoeducation program for chronic stable angina Can J Nurs Res 2007 39 48 65 17679585

[B8] McGillion MH Watt-Watson JH Kim J Graham A Learning by heart: a focused group study to determine the self-management learning needs of chronic stable angina patients Can J Cardiovasc Nurs 2004 14 12 22 15230024

[B9] MacDermott AFN Living with angina pectoris: a phenomenological study Eur J Cardiovasc Nurs 2002 1 265 272 10.1016/S1474-5151(02)00047-6 14622656

[B10] McGillion M Croxford R Watt-Watson J LeFort S Stevens B Coyte P Cost of illness for chronic stable angina patients enrolled in a self-management education trial Can J Cardiol 2008 24 759 764 10.1016/S0828-282X(08)70680-9 18841254 PMC2643155

[B11] Stewart S Murphy N Walker A McGuire A McMurray JJV The current cost of angina pectoris to the National Health Service in the UK Heart 2003 89 848 853 10.1136/heart.89.8.848 12860855 PMC1767798

[B12] Bandura A Self-efficacy: the exercise of control 1977 New York: WH Freeman

[B13] Bundy C Carroll D Wallace L Nagle R Psychological treatment of chronic stable angina pectoris Psychol Health 1994 10 69 77 10.1080/08870449408401937

[B14] Lewin B Cay E Todd I Soryal I Goodfield N Bloomfield P The angina management programme: a rehabilitation treatment Br J Cardiol 1995 1 221 226

[B15] Lewin RJP Furze G Robinson J Griffith K Wiseman S Pye M A randomized controlled trial of a self-management plan for patients with newly diagnosed angina Br J Gen Pract 2002 52 194 196 199–201 12030661 PMC1314238

[B16] Payne TJ Johnson CA Penzein DB Porzelius J Eldridge G Parisi S Chest pain self-management training for patients with coronary artery disease J Psychosom Res 1994 38 409 418 10.1016/0022-3999(94)90102-3 7965930

[B17] Gallacher JEJ Hopkinson CA Bennett ML Burr ML Elwood PC Effect of stress management on angina Psychol Health 1997 12 523 532 10.1080/08870449708406728

[B18] Ma W Teng Y Influence of cognitive and psychological intervention on negative emotion and severity of myocardial ischemia in patients with angina Chin J Clin Rehab 2005 24 25 27

[B19] McGillion M Watt-Watson J Stevens B LeFort S Coyte P Graham A Randomized controlled trial of a psychoeducation program for the self-management of chronic cardiac pain J Pain Symptom Manage 2008 36 126 140 10.1016/j.jpainsymman.2007.09.015 18395397

[B20] Furze G Cox H Morton V Chuang LH Lewin RJ Nelson P Randomized controlled trial of a lay-facilitated angina management programme J Adv Nurs 2012 68 2267 2279 10.1111/j.1365-2648.2011.05920.x 22229483 PMC3491702

[B21] Zetta S Smith K Jones M Allcoat P Sullivan F Evaluating the angina plan in patients admitted to hospital with angina: a randomized controlled trial Cardiovasc Ther 2011 29 112 124 10.1111/j.1755-5922.2009.00109.x 20041881

[B22] McGillion MH Watt-Watson JH Kim J Yamada J A systematic review of psychoeducational interventions for the management of chronic stable angina J Nurs Manag 2004 12 174 182 10.1111/j.1365-2834.2004.00472.x 15089955

[B23] McGillion M Arthur H Victor C Watt-Watson J Cosman T Effectiveness of psyhcoeducational interventions for improving symptoms, health-related quality of life, and psychological well being in patients with stable angina Curr Cardiol Rev 2008 4 1 11 10.2174/157340308783565393 19924272 PMC2774580

[B24] McGillion M Cook A Victor JC Carroll S Weston J Teoh K Effectiveness of percutaneous laser revascularization therapy for refractory angina Vasc Health Risk Manag 2010 6 735 747 20859544 10.2147/vhrm.s8222 PMC2941786

[B25] Higgins JPT Green S Cochrane Handbook for Systematic Reviews of Interventions Version 5.1.0 [updated March 2011] 2011 The Cochrane Collaboration Available from http://www.cochrane-handbook.org

[B26] Moher D Liberati A Tetzlaff J Altman DG The PRISMA Group Preferred reporting items for systematic reviews and meta-analyses: the PRISMA statement PLoS Med 2009 6 7 e1000097 doi: 10.1371/journal.pmed.1000097 10.1371/journal.pmed.1000097 19621072 PMC2707599

[B27] Review Manager (RevMan) [Computer program]. Version 5.2 2012 Copenhagen: The Nordic Cochrane Centre, The Cochrane Collaboration

[B28] Hozo SP Djulbegovic B Hozo I Estimating the mean and variance from the median, range, and the size of a sample BMC Med Res Methodol 2005 5 13 10.1186/1471-2288-5-13 15840177 PMC1097734

[B29] Tomlinson G Beyene J [P148] Imputing Summary Statistics for Meta-Analysis of Continuous Data 2004 Ottawa: 12th Cochrane Colloquium

[B30] Cohen J Statistical power analysis for the behavioral sciences 1988 2 Hillsdale: Lawrence Earlbaum Associates

[B31] DerSimonian R Laird N Meta-analysis in clinical trials Control Clin Trials 1986 7 177 188 10.1016/0197-2456(86)90046-2 3802833

[B32] Higgins JP Thompson SG Deeks JJ Altman DG Measuring inconsistency in meta-analyses BMJ 2003 327 557 560 10.1136/bmj.327.7414.557 12958120 PMC192859

[B33] Rucker G Schwarzer G Carpenter JR Schumacher M Undue reliance on I^2^ in assessing heterogeneity may mislead BMC Med Res Methodol 2008 8 79 10.1186/1471-2288-8-79 19036172 PMC2648991

[B34] Spertus JA Winder JA Dewhurst TA Deyo RA Prodzinski J McDonell M Development and evaluation of the Seattle Angina Questionnaire: a new functional status measure for coronary artery disease J Am Coll Cardiol 1995 25 333 341 10.1016/0735-1097(94)00397-9 7829785

[B35] Zigmond AS Snaith RP The hospital anxiety and depression scale Acta Psychiatr Scand 1983 67 361 370 10.1111/j.1600-0447.1983.tb09716.x 6880820

[B36] Dougherty C Dewhurst T Nichol P Spertus J Comparison of three quality of life instruments in stable angina pectoris: seattle angina questionnaire, short form health survey (SF-36), and quality of life index-cardiac version III J Clin Epidemiol 1998 51 569 575 10.1016/S0895-4356(98)00028-6 9674663

[B37] Parker JD Parker JO Stable angina pectoris: the medical management of symptomatic myocardial ischemia Can J Cardiol 2012 28 S70 S80 10.1016/j.cjca.2011.11.002 22424287

[B38] Jolicoeur EM Ohman EM Temple R Stockbridge N Smith S Mark D Clinical and research issues regarding chronic advanced coronary disease part II: trial design, outcomes, and regulator issues Am Heart J 2008 155 435 444 10.1016/j.ahj.2007.12.005 18294475

[B39] Whalley B Rees K Davies P Bennett P Ebrahim S Liu Z Psychological outcomes for coronary heart disease Cochrane Database Syst Rev 2011 Issue 8 CD002902 doi: 10.1002/14651858.CD002902.pub3 21833943 10.1002/14651858.CD002902.pub3

[B40] LeLorier J Grégoire G Benhaddad A Lapierre J Derderian F Discrepancies between meta-analyses and subsequent large randomized, controlled trials N Engl J Med 1997 337 536 542 10.1056/NEJM199708213370806 9262498

